# Bis(2-amino­pyridinium) 5,5′-disulfanediylbis(1,3,4-thia­diazole-2-thiol­ate) monohydrate

**DOI:** 10.1107/S1600536811008336

**Published:** 2011-03-12

**Authors:** Pusu Zhao, Zhiyan Guo, Hailian Xiao

**Affiliations:** a New Materials & Function Coordination Chemistry Laboratory, Qingdao University of Science and Technology, Qingdao Shandong 266042, People’s Republic of China; b College of Materials Science and Engineering, Qingdao University of Science & Technology, Qingdao 266042, People’s Republic of China

## Abstract

In the crystal of the title compound, 2C_5_H_7_N_2_^+^·C_4_N_4_S_6_^2−^·H_2_O, inter­molecular N—H⋯S and N—H⋯N hydrogen bonds link four cations and two dianions into a centrosymmetric cluster. The crystal packing is further consolidated by π–π inter­actions between the five- and six-membered rings of neighbouring clusters [centroid–centroid distances = 3.692 (3), 3.718 (3), 3.660 (3) and 3.696 (3) Å] and *via* O—H⋯N, O—H⋯S and N—H⋯O hydrogen bonds involving the uncoordinated water mol­ecules.

## Related literature

For general background to supra­molecular compounds, see: Rowsell & Yaghi (2005 ▶); Neville *et al.* (2008 ▶); Huang *et al.* (2007 ▶); Burchell *et al.* (2006 ▶). For related structures, see: Jebas *et al.* (2006 ▶); Jian *et al.* (2006 ▶); Banerjee *et al.* (2006 ▶); Moers *et al.* (2000 ▶).
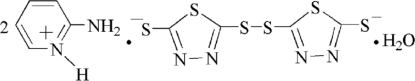


## Experimental

### 

#### Crystal data

2C_5_H_7_N_2_^+^·C_4_N_4_S_6_^2−^·H_2_O
*M* *_r_* = 504.71
Monoclinic, 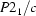

*a* = 7.3109 (15) Å
*b* = 14.112 (3) Å
*c* = 20.930 (4) Å
β = 98.13 (3)°
*V* = 2137.8 (7) Å^3^
*Z* = 4
Mo *K*α radiation
μ = 0.67 mm^−1^
*T* = 295 K
0.24 × 0.22 × 0.20 mm

#### Data collection

Enraf–Nonius CAD-4 diffractometer
9377 measured reflections
4911 independent reflections
3931 reflections with *I* > 2σ(*I*)
*R*_int_ = 0.023
3 standard reflections every 100 reflections  intensity decay: none

#### Refinement

*R*[*F* ^2^ > 2σ(*F* ^2^)] = 0.048
*wR*(*F* ^2^) = 0.086
*S* = 1.17
4911 reflections
270 parameters
H atoms treated by a mixture of independent and constrained refinement
Δρ_max_ = 0.55 e Å^−3^
Δρ_min_ = −0.26 e Å^−3^

### 

Data collection: *CAD-4 Software* (Enraf–Nonius, 1989 ▶); cell refinement: *CAD-4 Software*; data reduction: *NRCVAX* (Gabe *et al.*, 1989 ▶); program(s) used to solve structure: *SHELXS97* (Sheldrick, 2008 ▶); program(s) used to refine structure: *SHELXL97* (Sheldrick, 2008 ▶); molecular graphics: *SHELXTL* (Sheldrick, 2008 ▶); software used to prepare material for publication: *WinGX* (Farrugia, 1999 ▶).

## Supplementary Material

Crystal structure: contains datablocks global, I. DOI: 10.1107/S1600536811008336/cv5028sup1.cif

Structure factors: contains datablocks I. DOI: 10.1107/S1600536811008336/cv5028Isup2.hkl

Additional supplementary materials:  crystallographic information; 3D view; checkCIF report

## Figures and Tables

**Table 1:** Hydrogen-bond geometry (Å, °)

*D*—H⋯*A*	*D*—H	H⋯*A*	*D*⋯*A*	*D*—H⋯*A*
N1—H1*A*⋯S6	0.86	2.63	3.488 (3)	177
N2—H2*A*⋯N8	0.86	1.87	2.726 (3)	171
N3—H3*A*⋯S1	0.86	2.43	3.273 (3)	166
N4—H4*A*⋯N5	0.86	1.97	2.826 (3)	179
O1*W*—H2*W*1⋯N6	0.80 (4)	2.07 (4)	2.860 (3)	169 (4)
N1—H1*B*⋯O1*W*^i^	0.86	2.07	2.898 (3)	162
O1*W*—H1*W*1⋯S6^ii^	0.83 (4)	2.47 (4)	3.294 (3)	175 (3)
N3—H3*B*⋯S6^iii^	0.86	2.49	3.338 (2)	171

Symmetry codes: (i) 
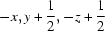
; (ii) 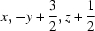; (iii) 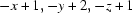.

**Fig. 1.**
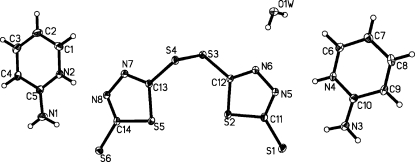
The structure of the title compound showing 30% probability displacement ellipsoids and the atom-numbering scheme.

**Fig. 2.**
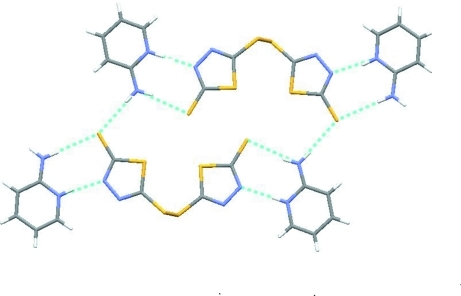
A hydrogen-bonded (dashed lines) centrosymmetric cluster in (I).

## supplementary crystallographic information

### Comment

Research on supramolecular compounds has become popular because of their potential applications in areas such as gas storage (Rowsell *et al.*, 2005), magnetics (Neville *et al.*, 2008) and optics (Huang *et al.*, 2007). Among many strategies for achieving supramolecular compounds with predefined structures, the choice and design of organic molecules as hydrogen-bond acceptors or donors are undoubtedly a key part of the construction of intriguing frameworks driven by hydrogen-bonding interactions (Burchell *et al.*, 2006).

However, in these hydrogen-bond supramolecular compounds, 2-amino-pyridine often only forms co-crystals with another organic acid, such as 3-aminobenzoic acid, naphthalene-1,5-disulfonic acid and nicotinic acid (Jebas *et al.*, 2006). It is less studied that the co-crystals are aggregated by 2-amino-pyridine with a non-acid compound. For example,non-acids compounds of saccharinate (Banerjee *et al.*, 2006) and bis(methanesulfonyl)amide (Moers *et al.*, 2000) have been used to form co-crystals with 2-aminopyridinium and their crystal structures have been reported.

Herein, we will give another report about the synthesis and chacterization of a 2:1 proton-transfer salt formed by 2-aminopyridinium with a non-acid compound of di(2-mercapto-1,3,4- thiadiazyl) disulfide and a water molecule.

Scheme I

The asymmetric unit contains one deprotonated di(2-mercapto-1,3,4-thiadiazyl) disulfide molecule, two protonated 2-amino-pyridine molecules and one water molecule. In the deprotonated di(2-mercapto-1,3,4- thiadiazyl) disulfide, two 2-mercapto-1,3,4-thiadiazyl groups are located at *cis*-position of the S3—S4 bond. In addition, these two 2-mercapto-1,3,4-thiadiazyl groups are in an opposite position with the dihedral angle between them being 6.84 (2)°. This geometry looks like that there is a *C*2 symmetric axis passing through the mid-point of the S3—S4 bond. The S3—S4 bond length is 2.06438 (11) Å, which is longer than that in dibenzothiazyl-disulfide [(2.027 (2) Å](Jian *et al.*, 2006). All of the other bond lengths and bond angles in the two pyridyl rings and two thiazolyl rings are in the normal range.

In the crystal lattice, there are eight kinds of hydrogen bonds (Table 2), five of which link two 2-mercapto-1,3,4- thiadiazyl disulfide and four 2-amino-pyridinium ions together to form a macrocycle unit and three of which related to the water molecule help to conjunct all of the macrocycles to build a three dimensional net works of the molecules.

As shown in Fig. 2, after accepting a proton from 2-mercapto-1,3,4-thiadiazyl disulfide, 2-amino-pyridinium ion has become a complete hydrogen bond donor. For example, each of the 2-amino-pyridinium ion containing N4 atom provides its three hydrogen bond donor sites such as N4—H4A, N3—H3A and N3—H3B to participate in building hydrogen bonds with two deprotonated 2-mercapto-1,3,4- thiadiazyl disulfide molecules, and finally to form three hydrogen bonds of N4—H4A···N5, N3—H3A···S1 and N3—H3B···S6. Each of the 2-amino-pyridinium ion containing N2 atom provides two hydrogen bond donor sites of N1—H1A and N2—H2A to construct hydrogen bonds with one deprotonated 2-mercapto-1,3,4- thiadiazyl disulfide molecule, and ultimately to form two hydrogen bonds of N1—H1A···S6 and N2—H2A···N8. Through above hydrogen bond connections, four 2-amino-pyridinium ions and two deprotonated 2-mercapto-1,3,4-thiadiazyl disulfide molecules are join together to give a macrocycle unit. Then, each three macrocycle units are linked together by a water molecule through three hydrogen bonds of N1—H1B···O1W, O1W-H1W1···S6 and O1W-H2W1···N6. Namely, the water molecule acts as two hydrogen bond donor as well as one hydrogen bond acceptor to join three macrocycle units together. Additionally, since the existence of two S3—S4 bonds, each macrocycle is bended to two layers and in each macrocycle unit, there are six hydrogen bond sites to link with six water molecules. Thus, all macrocycles are connected by water molecules to construct a three-dimensional architecture. On the other hand, among the pyridyl  and thiadiazyl rings, there are π···π stacking interactions (Table 2) which further stabilize the crystal packing.

### Experimental

Compound (I) was synthesized by heating together, for 20 min under reflux, 2-amino-pyridine (0.94 g, 10 mmol) and di(2-mercapto-1,3,4-thiadiazyl) disulfide (1.49 g, 5 mmol) in distilled water (30 ml). The colourless crystals were obtained after slow evaporation of the water solvent at room temperature.

### Refinement

The water H atoms were found in a difference Fourier map, and refined isotropically. All other H atoms were fixed geometrically (C—H 0.93 Å, N—H 0.86 Å), and treated as riding, with  *U*_iso_= 1.2 *U*_eq_ of the parent atom.

### Crystal data

**Table:**

2C_5_H_7_N_2_^+^·C_4_N_4_S_6_^2^^−^·H_2_O	*F*(000) = 1040
*M**_r_* = 504.71	*D*_x_ = 1.568 Mg m^−^^3^
Monoclinic, *P*2_1_/*c*	Mo *K*α radiation, λ = 0.71073 Å
Hall symbol: -P 2ybc	Cell parameters from 25 reflections
*a* = 7.3109 (15) Å	θ = 4–14°
*b* = 14.112 (3) Å	µ = 0.67 mm^−^^1^
*c* = 20.930 (4) Å	*T* = 295 K
β = 98.13 (3)°	Block, colourless
*V* = 2137.8 (7) Å^3^	0.24 × 0.22 × 0.20 mm
*Z* = 4	

### Data collection

**Table:**

Enraf–Nonius CAD-4 diffractometer	*R*_int_ = 0.023
Radiation source: fine-focus sealed tube	θ_max_ = 27.5°, θ_min_ = 1.8°
graphite	*h* = −9→9
ω scans	*k* = −18→17
9377 measured reflections	*l* = −27→27
4911 independent reflections	3 standard reflections every 100 reflections
3931 reflections with *I* > 2σ(*I*)	intensity decay: none

### Refinement

**Table:**

Refinement on *F*^2^	Primary atom site location: structure-invariant direct methods
Least-squares matrix: full	Secondary atom site location: difference Fourier map
*R*[*F*^2^ > 2σ(*F*^2^)] = 0.048	Hydrogen site location: inferred from neighbouring sites
*wR*(*F*^2^) = 0.086	H atoms treated by a mixture of independent and constrained refinement
*S* = 1.17	*w* = 1/[σ^2^(*F*_o_^2^) + (0.0278*P*)^2^ + 0.2825*P*] where *P* = (*F*_o_^2^ + 2*F*_c_^2^)/3
4911 reflections	(Δ/σ)_max_ = 0.001
270 parameters	Δρ_max_ = 0.55 e Å^−^^3^
0 restraints	Δρ_min_ = −0.26 e Å^−^^3^

### Special details

**Table:**

Geometry. All e.s.d.'s (except the e.s.d. in the dihedral angle between two l.s. planes) are estimated using the full covariance matrix. The cell e.s.d.'s are taken into account individually in the estimation of e.s.d.'s in distances, angles and torsion angles; correlations between e.s.d.'s in cell parameters are only used when they are defined by crystal symmetry. An approximate (isotropic) treatment of cell e.s.d.'s is used for estimating e.s.d.'s involving l.s. planes.
Refinement. Refinement of *F*^2^ against ALL reflections. The weighted *R*-factor *wR* and goodness of fit *S* are based on *F*^2^, conventional *R*-factors *R* are based on *F*, with *F* set to zero for negative *F*^2^. The threshold expression of *F*^2^ > σ(*F*^2^) is used only for calculating *R*-factors(gt) *etc*. and is not relevant to the choice of reflections for refinement. *R*-factors based on *F*^2^ are statistically about twice as large as those based on *F*, and *R*- factors based on ALL data will be even larger.

### Fractional atomic coordinates and isotropic or equivalent isotropic displacement parameters (Å^2^)

**Table:**

	*x*	*y*	*z*	*U*_iso_*/*U*_eq_	
S1	0.40901 (12)	0.94077 (5)	0.59925 (3)	0.0364 (2)	
S2	0.26262 (10)	0.84201 (4)	0.47444 (3)	0.02842 (17)	
S3	0.18855 (10)	0.64715 (4)	0.40985 (3)	0.02446 (15)	
S4	0.37606 (10)	0.66357 (4)	0.34565 (3)	0.02432 (15)	
S5	0.29173 (9)	0.86996 (4)	0.30034 (3)	0.02237 (15)	
S6	0.12212 (10)	0.98806 (4)	0.18451 (3)	0.02636 (16)	
N1	−0.1312 (3)	0.89557 (15)	0.04470 (11)	0.0380 (6)	
H1A	−0.0716	0.9175	0.0800	0.046*	
H1B	−0.1810	0.9339	0.0153	0.046*	
N2	−0.0676 (3)	0.74324 (14)	0.08195 (10)	0.0251 (5)	
H2A	−0.0060	0.7670	0.1163	0.030*	
N3	0.6664 (4)	0.84178 (15)	0.72187 (11)	0.0387 (6)	
H3A	0.6044	0.8593	0.6858	0.046*	
H3B	0.7133	0.8835	0.7493	0.046*	
N4	0.6138 (3)	0.68622 (14)	0.69089 (10)	0.0237 (5)	
H4A	0.5520	0.7064	0.6555	0.028*	
N5	0.4136 (3)	0.75310 (14)	0.57420 (9)	0.0231 (5)	
N6	0.3619 (3)	0.68528 (14)	0.52826 (9)	0.0231 (5)	
N7	0.1864 (3)	0.72209 (14)	0.23431 (10)	0.0254 (5)	
N8	0.1316 (3)	0.79840 (14)	0.19588 (9)	0.0247 (5)	
C1	−0.0815 (4)	0.64796 (18)	0.07686 (13)	0.0314 (6)	
H1C	−0.0260	0.6098	0.1104	0.038*	
C2	−0.1754 (4)	0.60765 (19)	0.02344 (14)	0.0349 (7)	
H2B	−0.1856	0.5421	0.0198	0.042*	
C3	−0.2571 (4)	0.66701 (19)	−0.02635 (13)	0.0322 (6)	
H3C	−0.3223	0.6405	−0.0634	0.039*	
C4	−0.2419 (4)	0.76228 (18)	−0.02103 (12)	0.0260 (6)	
H4B	−0.2945	0.8010	−0.0546	0.031*	
C5	−0.1462 (4)	0.80276 (17)	0.03563 (12)	0.0247 (6)	
C6	0.6319 (4)	0.59150 (17)	0.70022 (12)	0.0249 (6)	
H6B	0.5793	0.5503	0.6681	0.030*	
C7	0.7250 (4)	0.55568 (18)	0.75551 (12)	0.0274 (6)	
H7B	0.7364	0.4906	0.7620	0.033*	
C8	0.8036 (4)	0.62005 (19)	0.80266 (12)	0.0282 (6)	
H8B	0.8678	0.5972	0.8411	0.034*	
C9	0.7875 (4)	0.71541 (18)	0.79316 (12)	0.0266 (6)	
H9A	0.8410	0.7571	0.8248	0.032*	
C10	0.6895 (4)	0.75046 (18)	0.73512 (12)	0.0250 (6)	
C11	0.3711 (4)	0.84127 (17)	0.55446 (12)	0.0247 (5)	
C12	0.2803 (4)	0.72031 (16)	0.47427 (11)	0.0216 (5)	
C13	0.2721 (3)	0.74813 (16)	0.29047 (11)	0.0205 (5)	
C14	0.1749 (3)	0.88218 (17)	0.22216 (11)	0.0197 (5)	
O1W	0.3564 (3)	0.48901 (14)	0.56202 (10)	0.0342 (5)	
H2W1	0.371 (5)	0.542 (3)	0.5504 (17)	0.058 (12)*	
H1W1	0.293 (5)	0.498 (2)	0.5915 (18)	0.055 (12)*	

### Atomic displacement parameters (Å^2^)

**Table:**

	*U*^11^	*U*^22^	*U*^33^	*U*^12^	*U*^13^	*U*^23^
S1	0.0584 (6)	0.0233 (3)	0.0242 (4)	0.0096 (3)	−0.0051 (3)	−0.0068 (3)
S2	0.0413 (4)	0.0220 (3)	0.0193 (3)	0.0082 (3)	−0.0047 (3)	−0.0003 (2)
S3	0.0300 (4)	0.0230 (3)	0.0191 (3)	−0.0054 (3)	−0.0009 (3)	−0.0009 (2)
S4	0.0292 (4)	0.0247 (3)	0.0178 (3)	0.0056 (3)	−0.0008 (3)	0.0005 (2)
S5	0.0259 (4)	0.0225 (3)	0.0169 (3)	−0.0031 (3)	−0.0033 (3)	−0.0015 (2)
S6	0.0318 (4)	0.0221 (3)	0.0233 (3)	−0.0021 (3)	−0.0025 (3)	0.0033 (2)
N1	0.0514 (17)	0.0233 (11)	0.0325 (13)	−0.0013 (11)	−0.0176 (12)	0.0000 (9)
N2	0.0286 (13)	0.0262 (11)	0.0180 (11)	−0.0024 (9)	−0.0053 (9)	−0.0025 (8)
N3	0.0507 (17)	0.0279 (12)	0.0316 (13)	−0.0065 (11)	−0.0146 (12)	−0.0018 (9)
N4	0.0251 (13)	0.0281 (11)	0.0159 (10)	−0.0007 (9)	−0.0035 (9)	−0.0007 (8)
N5	0.0300 (13)	0.0223 (10)	0.0157 (10)	0.0003 (9)	−0.0011 (9)	−0.0004 (8)
N6	0.0275 (13)	0.0221 (10)	0.0187 (11)	−0.0015 (9)	−0.0004 (9)	0.0005 (8)
N7	0.0345 (14)	0.0214 (11)	0.0184 (10)	0.0005 (9)	−0.0030 (10)	−0.0006 (8)
N8	0.0325 (14)	0.0220 (10)	0.0170 (11)	−0.0012 (9)	−0.0052 (9)	−0.0003 (8)
C1	0.0382 (18)	0.0242 (14)	0.0312 (15)	0.0003 (12)	0.0024 (13)	0.0047 (11)
C2	0.0412 (19)	0.0233 (13)	0.0400 (17)	−0.0050 (12)	0.0053 (14)	−0.0069 (11)
C3	0.0278 (16)	0.0399 (16)	0.0282 (14)	−0.0052 (12)	0.0010 (12)	−0.0121 (11)
C4	0.0232 (15)	0.0342 (14)	0.0191 (13)	0.0012 (11)	−0.0023 (11)	−0.0019 (10)
C5	0.0228 (15)	0.0274 (13)	0.0227 (13)	−0.0019 (11)	−0.0012 (11)	−0.0026 (10)
C6	0.0241 (15)	0.0272 (13)	0.0235 (13)	−0.0001 (11)	0.0032 (11)	−0.0044 (10)
C7	0.0249 (15)	0.0290 (13)	0.0286 (14)	0.0047 (11)	0.0050 (12)	0.0023 (11)
C8	0.0204 (14)	0.0443 (16)	0.0200 (13)	0.0036 (12)	0.0031 (11)	0.0049 (11)
C9	0.0226 (15)	0.0355 (15)	0.0203 (13)	−0.0033 (11)	−0.0021 (11)	−0.0044 (10)
C10	0.0232 (15)	0.0266 (13)	0.0248 (13)	−0.0041 (11)	0.0017 (11)	−0.0042 (10)
C11	0.0288 (15)	0.0274 (13)	0.0174 (12)	0.0038 (11)	0.0016 (11)	−0.0008 (10)
C12	0.0247 (14)	0.0212 (12)	0.0180 (12)	−0.0013 (10)	0.0002 (10)	−0.0030 (9)
C13	0.0222 (14)	0.0215 (12)	0.0174 (12)	0.0018 (10)	0.0009 (10)	0.0008 (9)
C14	0.0189 (13)	0.0248 (12)	0.0152 (12)	−0.0007 (10)	0.0013 (10)	0.0005 (9)
O1W	0.0491 (15)	0.0229 (11)	0.0295 (12)	−0.0011 (9)	0.0022 (10)	0.0002 (8)

### Geometric parameters (Å, °)

**Table:**

S1—C11	1.689 (3)	N6—C12	1.299 (3)
S2—C12	1.722 (2)	N7—C13	1.304 (3)
S2—C11	1.749 (3)	N7—N8	1.370 (3)
S3—C12	1.754 (2)	N8—C14	1.324 (3)
S3—S4	2.0638 (11)	C1—C2	1.352 (4)
S4—C13	1.757 (2)	C1—H1C	0.9300
S5—C13	1.735 (2)	C2—C3	1.403 (4)
S5—C14	1.744 (2)	C2—H2B	0.9300
S6—C14	1.708 (2)	C3—C4	1.352 (4)
N1—C5	1.326 (3)	C3—H3C	0.9300
N1—H1A	0.8600	C4—C5	1.410 (3)
N1—H1B	0.8600	C4—H4B	0.9300
N2—C5	1.348 (3)	C6—C7	1.355 (4)
N2—C1	1.351 (3)	C6—H6B	0.9300
N2—H2A	0.8600	C7—C8	1.403 (4)
N3—C10	1.324 (3)	C7—H7B	0.9300
N3—H3A	0.8600	C8—C9	1.363 (4)
N3—H3B	0.8600	C8—H8B	0.9300
N4—C6	1.355 (3)	C9—C10	1.410 (4)
N4—C10	1.356 (3)	C9—H9A	0.9300
N4—H4A	0.8600	O1W—H2W1	0.80 (4)
N5—C11	1.334 (3)	O1W—H1W1	0.83 (4)
N5—N6	1.371 (3)		
			
Cg1···Cg3^i^	3.692 (3)	Cg2···Cg4^iii^	3.660 (3)
Cg1···Cg3^ii^	3.718 (3)	Cg2···Cg4^iv^	3.696 (3)
			
C12—S2—C11	88.39 (12)	N1—C5—N2	119.6 (2)
C12—S3—S4	102.56 (9)	N1—C5—C4	122.8 (2)
C13—S4—S3	103.82 (9)	N2—C5—C4	117.6 (2)
C13—S5—C14	88.00 (11)	N4—C6—C7	121.2 (2)
C5—N1—H1A	120.0	N4—C6—H6B	119.4
C5—N1—H1B	120.0	C7—C6—H6B	119.4
H1A—N1—H1B	120.0	C6—C7—C8	117.7 (2)
C5—N2—C1	122.9 (2)	C6—C7—H7B	121.1
C5—N2—H2A	118.5	C8—C7—H7B	121.1
C1—N2—H2A	118.5	C9—C8—C7	121.2 (2)
C10—N3—H3A	120.0	C9—C8—H8B	119.4
C10—N3—H3B	120.0	C7—C8—H8B	119.4
H3A—N3—H3B	120.0	C8—C9—C10	119.7 (2)
C6—N4—C10	122.6 (2)	C8—C9—H9A	120.2
C6—N4—H4A	118.7	C10—C9—H9A	120.2
C10—N4—H4A	118.7	N3—C10—N4	118.7 (2)
C11—N5—N6	113.89 (19)	N3—C10—C9	123.8 (2)
C12—N6—N5	113.05 (19)	N4—C10—C9	117.5 (2)
C13—N7—N8	111.79 (19)	N5—C11—S1	126.22 (19)
C14—N8—N7	115.17 (19)	N5—C11—S2	110.88 (18)
N2—C1—C2	120.5 (3)	S1—C11—S2	122.88 (15)
N2—C1—H1C	119.8	N6—C12—S2	113.78 (18)
C2—C1—H1C	119.8	N6—C12—S3	121.56 (18)
C1—C2—C3	118.4 (2)	S2—C12—S3	124.59 (14)
C1—C2—H2B	120.8	N7—C13—S5	114.06 (17)
C3—C2—H2B	120.8	N7—C13—S4	120.60 (18)
C4—C3—C2	120.7 (3)	S5—C13—S4	125.02 (14)
C4—C3—H3C	119.6	N8—C14—S6	124.38 (18)
C2—C3—H3C	119.6	N8—C14—S5	110.99 (17)
C3—C4—C5	119.8 (2)	S6—C14—S5	124.63 (14)
C3—C4—H4B	120.1	H2W1—O1W—H1W1	102 (3)
C5—C4—H4B	120.1		
			
C12—S3—S4—C13	−98.27 (12)	N6—N5—C11—S2	−0.5 (3)
C11—N5—N6—C12	−0.4 (3)	C12—S2—C11—N5	0.9 (2)
C13—N7—N8—C14	0.1 (3)	C12—S2—C11—S1	−177.71 (19)
C5—N2—C1—C2	0.7 (4)	N5—N6—C12—S2	1.1 (3)
N2—C1—C2—C3	0.2 (4)	N5—N6—C12—S3	−175.97 (18)
C1—C2—C3—C4	0.1 (5)	C11—S2—C12—N6	−1.1 (2)
C2—C3—C4—C5	−1.2 (4)	C11—S2—C12—S3	175.82 (19)
C1—N2—C5—N1	178.1 (3)	S4—S3—C12—N6	−106.0 (2)
C1—N2—C5—C4	−1.9 (4)	S4—S3—C12—S2	77.26 (17)
C3—C4—C5—N1	−177.9 (3)	N8—N7—C13—S5	0.1 (3)
C3—C4—C5—N2	2.1 (4)	N8—N7—C13—S4	−173.73 (18)
C10—N4—C6—C7	1.2 (4)	C14—S5—C13—N7	−0.1 (2)
N4—C6—C7—C8	−0.5 (4)	C14—S5—C13—S4	173.34 (18)
C6—C7—C8—C9	−0.2 (4)	S3—S4—C13—N7	−100.7 (2)
C7—C8—C9—C10	0.3 (4)	S3—S4—C13—S5	86.25 (17)
C6—N4—C10—N3	179.5 (2)	N7—N8—C14—S6	−179.89 (19)
C6—N4—C10—C9	−1.1 (4)	N7—N8—C14—S5	−0.2 (3)
C8—C9—C10—N3	179.7 (3)	C13—S5—C14—N8	0.2 (2)
C8—C9—C10—N4	0.3 (4)	C13—S5—C14—S6	179.88 (18)
N6—N5—C11—S1	178.1 (2)		

Symmetry codes: (i) *x*, −*y*+3/2, *z*−1/2; (ii) *x*−1, −*y*+3/2, *z*−1/2; (iii) *x*+1, −*y*+3/2, *z*+1/2; (iv) *x*, −*y*+3/2, *z*+1/2.

### Hydrogen-bond geometry (Å, °)

**Table:**

*D*—H···*A*	*D*—H	H···*A*	*D*···*A*	*D*—H···*A*
N1—H1A···S6	0.86	2.63	3.488 (3)	177
N2—H2A···N8	0.86	1.87	2.726 (3)	171
N3—H3A···S1	0.86	2.43	3.273 (3)	166
N4—H4A···N5	0.86	1.97	2.826 (3)	179
O1W—H2W1···N6	0.80 (4)	2.07 (4)	2.860 (3)	169 (4)
N1—H1B···O1W^v^	0.86	2.07	2.898 (3)	162
O1W—H1W1···S6^iv^	0.83 (4)	2.47 (4)	3.294 (3)	175 (3)
N3—H3B···S6^vi^	0.86	2.49	3.338 (2)	171

Symmetry codes:  (v) −*x*, *y*+1/2, −*z*+1/2; (iv) *x*, −*y*+3/2, *z*+1/2; (vi) −*x*+1, −*y*+2, −*z*+1.
